# DNA Double Strand Break and Response Fluorescent Assays: Choices and Interpretation

**DOI:** 10.3390/ijms25042227

**Published:** 2024-02-13

**Authors:** Jake Atkinson, Eva Bezak, Hien Le, Ivan Kempson

**Affiliations:** 1 Future Industries Institute, University of South Australia, Mawson Lakes, SA 5095, Australia; jake.atkinson@mymail.unisa.edu.au; 2 UniSA Allied Health and Human Performance, University of South Australia, Adelaide, SA 5095, Australia; eva.bezak@unisa.edu.au (E.B.);; 3 Department of Physics, University of Adelaide, North Terrace, Adelaide, SA 5005, Australia; 4 Department of Radiation Oncology, Royal Adelaide Hospital, Adelaide, SA 5000, Australia

**Keywords:** microscopy, non-homologous end joining (NHEJ), homologous-recombination (HR), γH2AX, p53 Binding Protein 1 (53BP1), foci, pathway choice, repair

## Abstract

Accurately characterizing DNA double-stranded breaks (DSBs) and understanding the DNA damage response (DDR) is crucial for assessing cellular genotoxicity, maintaining genomic integrity, and advancing gene editing technologies. Immunofluorescence-based techniques have proven to be invaluable for quantifying and visualizing DSB repair, providing valuable insights into cellular repair processes. However, the selection of appropriate markers for analysis can be challenging due to the intricate nature of DSB repair mechanisms, often leading to ambiguous interpretations. This comprehensively summarizes the significance of immunofluorescence-based techniques, with their capacity for spatiotemporal visualization, in elucidating complex DDR processes. By evaluating the strengths and limitations of different markers, we identify where they are most relevant chronologically from DSB detection to repair, better contextualizing what each assay represents at a molecular level. This is valuable for identifying biases associated with each assay and facilitates accurate data interpretation. This review aims to improve the precision of DSB quantification, deepen the understanding of DDR processes, assay biases, and pathway choices, and provide practical guidance on marker selection. Each assay offers a unique perspective of the underlying processes, underscoring the need to select markers that are best suited to specific research objectives.

## 1. Introduction

Double-stranded breaks are one of the most harmful forms of DNA damage that cells can sustain. They are characterized by breakage of the sugar-phosphate backbone on opposing strands of a DNA molecule, occurring within a proximity of ~10–20 base pairs [1]. The cellular response to this form of damage is rapid and prone to stringent regulation, as the presence of DSBs results in a variety of degradative effects that severely impact cell survival. These effects include the destabilization of genomic DNA via chromosomal translocations, inversions, and deletions [2]. Although these may result in cell death, some deleterious mutations may confer beneficial clonal advantages, resulting in the uncontrolled regulation of cell growth and progression to cancerous phenotypes. Tumorigenic cells can thus withstand a higher frequency of DSBs due to the overexpression of oncogenes and contain mutations within key factors of the DNA damage response [3]. In contrast, normal cell apoptosis can be triggered with just one unrepaired DSB if an essential locus is disrupted [4].

There are a variety of genotoxic factors that pose a threat to DNA integrity, both natural and artificial. For instance, radiation exposure comes from terrestrial sources, including naturally occurring radioisotopes in soil and water, and from bioaccumulation within the ecosystem’s flora and fauna. The inhalation of radon, a naturally occurring radioactive gas, contributes towards approximately 50% of the annual background radiation exposure for the global population [5,6]. Ultraviolet-B wavelengths in sunlight, which have health benefits in facilitating the production of endorphins and vitamin D, are also the principal risk factor for developing skin cancer [7]. With the advent of interplanetary and commercial space travel, understanding the impact of cosmic radiation is crucial for the wellbeing of astronauts during long missions [8,9]. On the other hand, manmade genotoxic sources include pesticides, alcohol, tobacco smoke, and certain medications. Despite the potential harm posed by radiation, it is a crucial tool in the medical field for both diagnosing and treating illness. This includes providing detailed images of the body through X-ray imaging and computed tomography and treating cancer through radiation therapy, which over 50% of patients in developed countries undergo [10]. Given that people are constantly exposed to genotoxic sources through their lifetime, it is crucial to accurately characterize and assess the damage caused and how cells act in response.

The repair of DNA DSBs occurs via a number of pathways, each comprising the intricate timing of sensing damage, recruiting appropriate repair machinery to the breakage site, and ultimately repair. Several variables determine the pathway responsible for repairing DSBs. Cellular conditions, including the cell cycle phase, availability of DNA repair proteins, and severity of damage, all contribute towards which factors are utilized to repair DNA lesions [1]. To validate the presence of DSBs experimentally, many current studies utilize immunofluorescence techniques to target DDR proteins that are activated in response to DSB presence. Established markers, including the commonly used γH2AX and 53BP1, typify the early steps in the initiation of the DDR and are often assumed to be reliable indications of DSB presence [11]. However, fluorescence signals generated by these markers are not a direct indication of DSBs themselves. Rather, the presence of these markers signifies the initiation of DSB repair and not the presence of the actual breakage sites [12]. Tagging these proteins is an indirect method of DSB detection [13], but it is still extremely useful for identifying relationships among damage formation, repair efficacy, and repair kinetics. However, detecting actual DSBs, rather than markers of their repair, with a high degree of reliability is still challenging. As DSB repair is complex and prone to error [1], its detection assays are influenced by multiple factors. These include missing DSBs that have already been repaired by the DDR, competition for binding with endogenous DDR factors [14], and a lack of sensitivity, all of which can produce false negatives. Conversely, issues also arise with techniques that lack specificity. For instance, the terminal transferase dUTP nick end-labeling (TUNEL) assay is commonly used to assess the extent of DNA fragmentation to identify cells undergoing apoptotic cell death [15]. This includes the labeling of DSBs, to which fluorescently labeled nucleotides are conjugated to free 3′-OH DNA ends at breakage sites. However, it is limited in its specificity as it is also capable of binding single-strand DNA (ssDNA) nicks or single-stranded breaks (SSBs), which occur at a frequency of ~10,000 breakages per day per cell [16]. This results in pan-nuclear staining, which is incapable of resolving discrete DSB foci, but useful in the binary determination of whether cells are apoptotic/necrotic. This can also occur when staining cells exposed to very high radiation doses [17], which is another important consideration when performing immunofluorescence assays.

Other classic methods of DNA damage detection include the comet assay, where fragmented DNA is separated from intact DNA through gel electrophoresis. The greater the extent of DNA fragmentation within a cell, the greater the proportion DNA that will reside within the comet “tail” formed versus the intact DNA “head” [18]. The alkaline-halo assay similarly measures the ratio of fragmentated DNA, but instead damaged DNA will spread outwards to form a halo structure around the nuclear cage, whereas intact DNA will not diffuse [19,20]. These techniques have also been combined with Fluorescence In-Situ Hybridization (FISH). Notably, DBD-FISH utilizes a whole-genome probe in combination with comet to simultaneously detect SSBs and DSBs, with Fernandez et al. proposing that the average FISH probe intensity and comet area correlate with the presence of each lesion, respectively [21]. Akin to TUNEL, these techniques are limited as although they can quantify the proportion of DNA fragmentation that occurs on a single-cell level, they lack the ability to visualize individual DSB sites or repair factors and thus will not be discussed in this review. 

**Figure 1:**
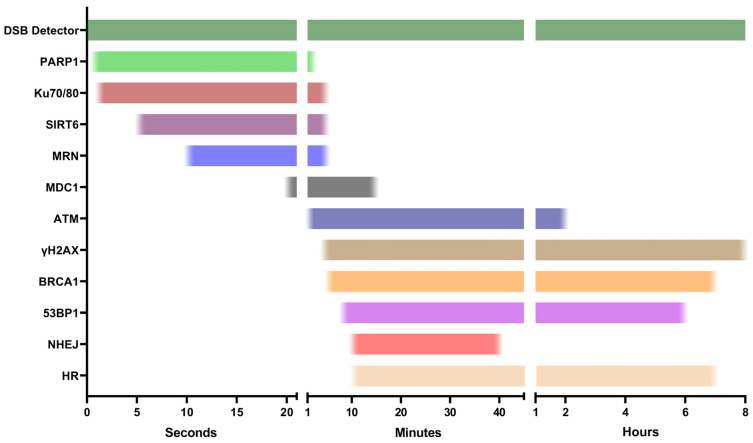
Timescale depicting the temporal kinetics of DSB detectors and key DDR proteins at peak activity throughout the DSB repair process after genotoxic exposures, based upon previous fluorescence imaging studies. DSB detectors utilizing exogenous factors are theoretically capable of DSB detection across all timepoints where DSBs are present (see DSB detector assays). Endogenous markers are sequentially recruited to the breakage site in a signaling cascade, resulting in the accumulation of repair factors to the DSB site. Some factors bind transiently over the course of minutes (PARP1 [22], Ku70/80 [23], SIRT6 [23], MRN [22], MDC1 [24]), whilst others persist and remain detectable hours post-insult (ATM [25,26], γH2AX [27], BRCA1 [28], 53BP1 [29,30], NHEJ/HR [31]).

Ultimately, different markers are required for assays depending on the specific research question being investigated. Knowing the correct context and circumstances for which specific markers should be used is critical in drawing accurate conclusions from DDR data. Therefore, characterizing double-stranded breaks allows us to better evaluate multiple forms of genotoxicity and the impact they have on cell genomic integrity and to obtain a more reliable gauge of the severity they present to cellular function. The intrinsic links among DSB and DDR factor accumulation, the cell cycle, cell death, and the biological impact of genotoxic insults, means that characterizing DSBs not only allows us to better understand the extent of genotoxic insults, but also in accurately understanding the mechanisms responsible for DSB repair. Thus, this review considers the formation of a DSB and then in chronological order steps through the DSB detection and repair pathways with respect to fluorescence-based assays that can identify specific aspects of the DNA damage formation and response (Figure 1). By doing so, the review identifies what individual assays technically represent at a molecular level. This is valuable for identifying biases associated with each assay and facilitates accurate data interpretation (Table 1). It also enables decision making for conducting the best assays aimed at specific research questions. The assays included here encompass both common and novel fluorescence-based techniques and the advantages and disadvantages each present in identifying DSB repair or DSBs specifically. The aim of this review is to provide researchers with the tools required to make informed decisions about which assays are best suited to their specific needs, leading to more insightful and reliable data.

## 2. DSB Detector Assays

A DSB detector can be defined as a biomolecular marker utilized to identify and quantify DSBs. These are often exogenous molecules that function by binding to one or both ends of a DSB and generate a signal that can be visualized via fluorescence microscopy when successfully bound. Currently, there are two main methods. The first is by utilizing proteins that typically possess DSB-end binding capabilities, conjugated with a fluorescent protein. The other is to incorporate oligonucleotides at the 3′-OH ends of a DSB, which can be tagged with probes that emit fluorescence signals when in proximity. Assessing DSBs via methods mentioned in this section allow for the direct quantification of breaks, without reliance on marking endogenous DDR factors. This allows for the number of breaks to be determined without the multitude of factors that can influence DDR factor expression, thereby providing an unambiguous understanding of the extent of genotoxic damage in cells.

### 2.1. GamGFP

**Figure 2:**
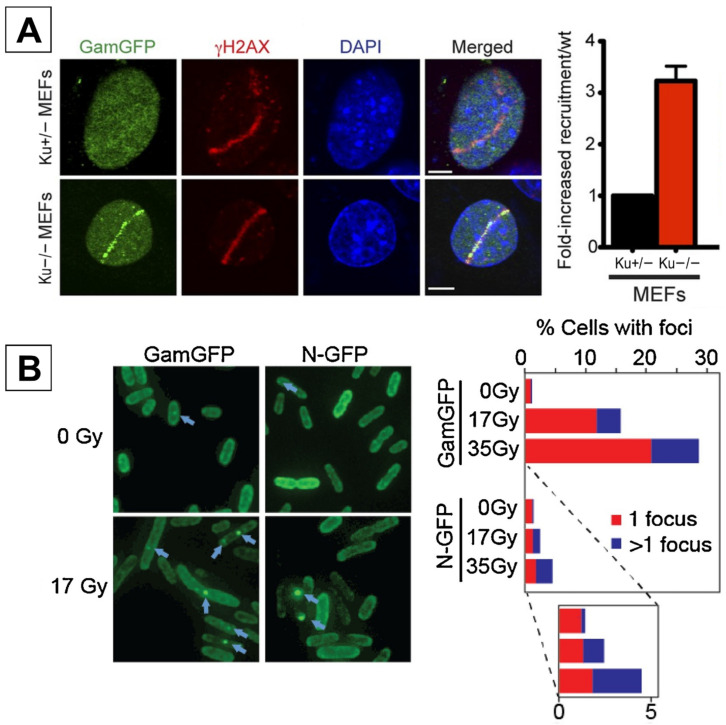
(**A**) Co-localization of GamGFP with the DDR marker γH2AX after laser-induced damage in either Ku+/− or Ku−/− MEFs. A marked reduction in GamGFP foci is observed in Ku+/− MEFs, where Ku acts as a competitive inhibitor of GamGFP [14]. Reprinted with permission from Ref. [14]. 2013, eLife under the CC BY 4.0 Deed|Attribution 4.0 International|Creative Commons. (**B**) Comparison of GamGFP and N-GFP foci formation in *E. coli* cells after exposure to 0, 17, or 35 Gy X-rays. Approximately 30% of GamGFP cells presented foci at 35 Gy compared to 5% for N-GFP [37]. Reprinted with permission from [37]. 2018, DNA Repair|Vol 72, Pages 1–116 |ScienceDirect.com by Elsevier.

In 2013, Shee et al. reported a new fluorescent marker to identify DSBs, desiring a direct method of detection [14]. They produced a doxycycline-inducible plasmid containing DSB binding protein “Gam”, derived from bacteriophage Mu, bound to green fluorescent protein (GFP). This protein is an ortholog to the NHEJ Ku proteins used in both bacterial and mammalian cells [32], but its function when used by phage Mu differs substantially from its DDR roots. Upon phage infection, Gam’s role is to prevent the action of cellular exonucleases by binding to the ends of linear phage DNA, thereby protecting it from degradation. Mu Gam is capable of binding to either blunt- or sticky-ended DSBs [33], making it a prime candidate for a DSB marker. GamGFP binds directly to the site of the DSB lesion, allowing for the observation of discrete GFP foci where DSB ends are present. According to their study, GamGFP foci appear to co-localize with γH2AX, but not extensively with 53BP1. Using Ku80-deficient mouse embryonic fibroblasts (MEFs) after laser micro-irradiation, co-localization between Gam and 53BP1 was 31%. Note that although laser-microirradiation does induce DSBs, a variety of other genomic lesions are also formed, which may cause DSB repair factors to accumulate due to other types of DNA lesions [34]. Shee et al. suggest that this is due to GamGFP detecting DSBs with higher speed and specificity compared to 53BP1. However, one major drawback to this technique is that the efficiency of binding GamGFP to damage sites within mammalian cells is lowered due to competition with endogenous Ku. A 2-fold increase in GamGFP foci was observed in Ku−/− MEFs when compared to a Ku+/− knockout (Figure 2A) [14].

Therefore, whilst this marker is useful in specifically marking DSBs, its competition to bind to DSBs with Ku means it underestimates the total number of DSBs, and it is difficult to utilize without first producing Ku knockouts prior to experimentation. In terms of complexity, this assay involves production of a GamGFP fusion gene in an inducible plasmid and then transfection or transformation into a mammalian or bacterial cell line of interest, respectively. Issues may arise in optimizing transfection efficiency, with different cell lines varying greatly in this regard [35]. However, compared to other techniques listed in this review herein, the concept of utilizing GamGFP as a DSB marker is relatively simple and reliable, most effective for use in bacterial studies, but under-represents DSBs in mammalian models. An example of the type of experiment where this assay would be appropriate is Nath et al., who previously utilized GamGFP to evaluate DSB formation in *Escherichia coli* (*E. coli*) in response to fluoroquinolone antibiotic treatment [36].

Kotlajich et al. also sought to produce a similar DSB end-labeling GFP fusion protein to Gam-GFP [37]. They utilized the N protein of phage Mu, which has been previously reported to bind Mu phage DNA non-covalently in a circular complex, and thus investigated whether this protein might possess DSB ending properties. Foci formation was compared between N- and Gam-GFP in *E. coli* exposed to either phleomycin (a radiomimetic agent), enzymatic cleavage, or X-rays. The proportion of cells with N-GFP foci in response to phleomycin treatment was approximately half that of Gam-GFP, and 4-to-6 times fewer in response to I-SceI restriction enzyme digestion or X-rays (Figure 2B).

Although they found that N-GFP was capable of binding to DSB sites, data from the phleomycin treatments also suggested it could label “non-DSB” DNA damage. Unlabeled Gam was also produced via an expression vector to determine whether the binding of N-GFP was competitively inhibited by Gam occupying DSB ends. N-GFP foci formation was found to remain consistent regardless of Gam production, implying that N-GFP identifies DNA damage distinct from DSBs (presumably SSBs) [37].

### 2.2. DI-PLA

**Figure 3:**
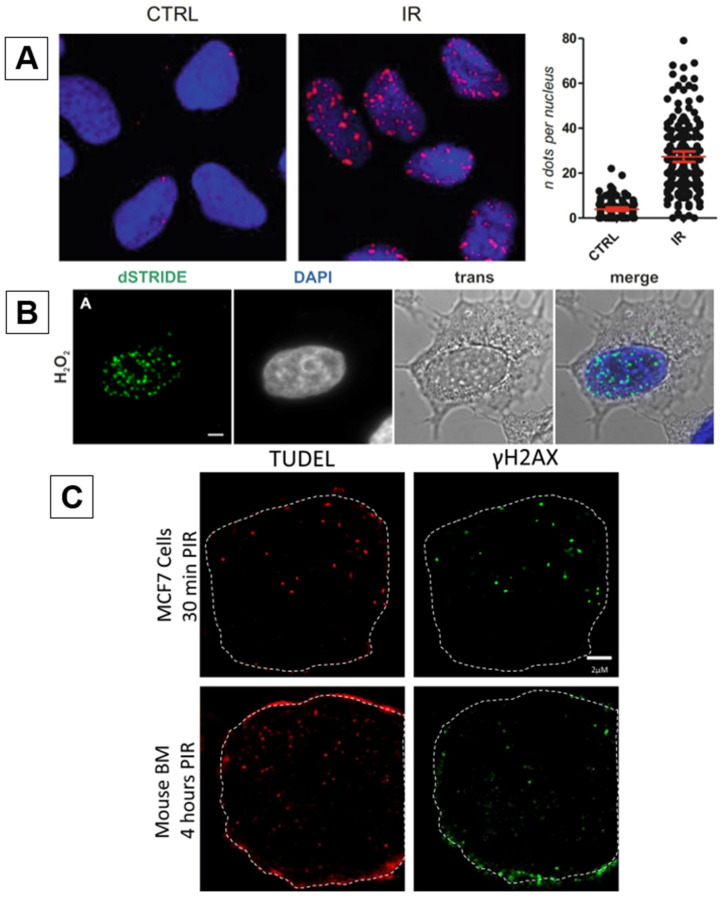

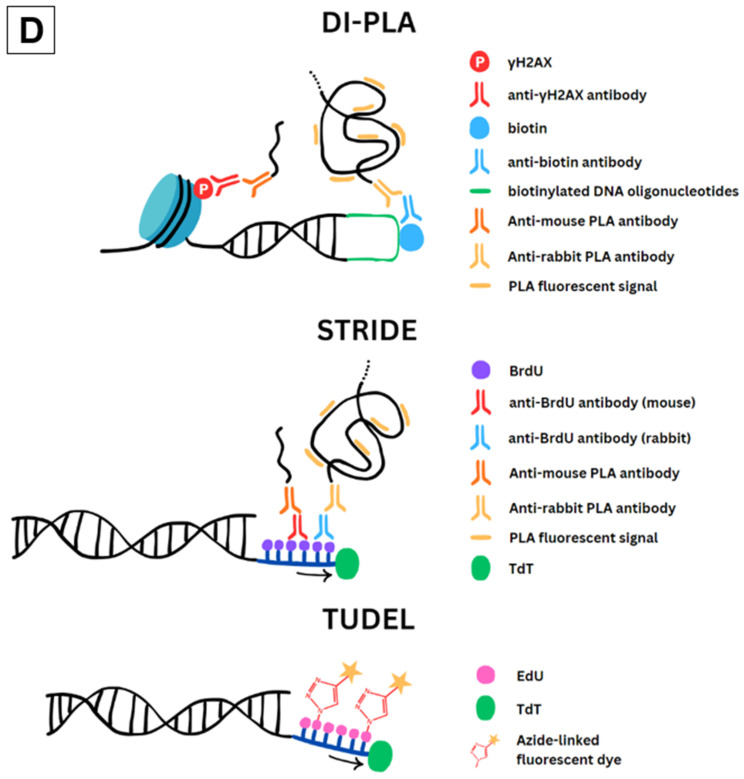
(**A**) U2OS cells treated with or without an inducible restriction enzyme (AsiSI). DI-PLA (yellow) was employed with antibodies against biotin (biotinylated linker nucleotides) and γH2AX. Nuclear DNA counterstained with DAPI (blue) [38,39]. Reprinted with permission from Refs. [38,39]. 2017, Springer Nature. (**B**) HeLa cells exposed to 4 mM hydrogen peroxide and stained with dSTRIDE (green) and DAPI (blue). dSTRIDE foci are representative of the tagging of free 3′-OH DNA ends within both the nucleus and the cytoplasm by [40]. Reprinted with permission from Ref. [40]. 2019, Oxford University Press on behalf of Nucleic Acids Research. (**C**) MCF7 cells exposed to 6 Gy from a Cs-137 source, fixed 30 min post-irradiation (PIR). Cells were stained with TUDEL and anti-γH2AX antibodies. TUDEL (red) co-localizes with γH2AX foci (green) within the nucleus (dotted line represents approximate border) [41]. Reproduced with permission from Ref. [41]. 2022, Springer Nature. (**D**) Diagrammatic representation of the differences among the DI-PLA, STRIDE, and TUDEL DSB detection assays. DI-PLA and STRIDE share similarities in employing a proximity ligation assay (PLA) reaction to produce fluorescence, whereas STRIDE and TUDEL share similarity in utilizing TdT to incorporate the oligonucleotide analogues 5-bromo-2′-deoxyuridine (BrdU) and ethynyl-dUTP (EdU), respectively.

In labeling DSB ends, three similar techniques have been produced, which rely upon three basic steps ensuring the specific labeling of double stranded breaks. These being the conjugation of a nucleotide analogue to mark free DNA ends, labeling of these DSB end-bound analogues with fluorescent marker-bound antibodies, and production of a fluorescent signal when these antibodies are in proximity to each other. The first of these techniques was reported in 2017 by Galbiati et al., termed the “DNA damage In situ ligation followed by Proximity Ligation Assay” (DI-PLA) [38]. Cells are first paraformaldehyde-fixed and permeabilized, followed by treatment with T4 DNA polymerase to blunt the DSB DNA ends. These DNA ends are then tagged by biotinylated nucleotides using T4 ligase at the site of a break. Following this, primary antibodies against both the biotinylated nucleotides and a proximal complex (e.g., γH2AX, core histone) are added. As the presence of an individual biotin molecule does not produce a sufficiently strong signal, a proximity ligation assay is performed. Secondary antibodies, directed against each primary antibody, contain conjugated oligonucleotides to facilitate a rolling circle amplification reaction that initiates when the biotin-tagged DSB and proximal DDR protein are within range (approx. 40 nm). This produces a strong fluorescent signal and distinct foci when observed via immunofluorescence microscopy (Figure 3A) [38]. 

In addition to identifying DSBs in proximity to DDR factors, this technique could also be adapted to identify breakages in proximity to other proteins, such as centromeres or telomeres, to further increase the specificity of where damage is identified. This is potentially problematic when concerning DDR factors, such as γH2AX, where changes in DDR activity will influence the number of DSBs detected, decreasing the accuracy of DSB quantification. Further discussion and comparison with STRIDE and TUDEL are provided at the end of this section.

### 2.3. STRIDE

In 2020, Kordon et al. published a new fluorescence-based technique for detecting DSBs called SensiTive Recognition of Individual DNA Ends (STRIDE), which when modified allows for the in situ detection of either single or double-strand breaks [40]. The initial steps of the STRIDE DSB detection method share similarities to the TUNEL assay [42]. First, the nucleotide analogue 5-bromo-2′-deoxyuridine (BrdU) is conjugated to free 3′-OH DNA ends enzymatically via terminal deoxynucleotide transferase (TdT). Two primary antibodies, derived from separate hosts and specific to BrdU, are then bound. Subsequently, two secondary antibodies, each specific to one of the primary antibodies, are also attached. Each secondary antibody is conjugated with its own set of oligonucleotides, which are then ligated to hybridizing connector oligonucleotides, forming a circular DNA template. Akin to DI-PLA, a rolling circle amplification reaction takes place, with one of the secondary antibody’s attached oligonucleotides acting as a primer for DNA polymerase. This newly synthesized concatemeric sequence allows for the hybridization of fluorescently labeled probes to these sequences, producing a strong amplified signal where DSBs are present (Figure 3B) [43]. 

The practicalities of the STRIDE technique include its ability to tag all double-strand break DNA breakages throughout the cell. Often when characterizing genotoxicity, measurement of the nuclear response is used as a measure of damage severity. Mitochondrial DSBs also cause stress through the triggering of p21/p53 cell cycle arrest, resulting in impaired proliferation [44]. As STRIDE is capable of labeling 3′-OH DNA ends, STRIDE can also identify mitochondrial DNA damage, present as discrete foci within the cytoplasm. One caveat this presents is that incorporation may occur with free cytoplasmic DNA fragments that are not damage-associated, decreasing specificity.

### 2.4. TUDEL

Building on the principles of the TUNEL assay and sharing aspects of both STRIDE and DI-PLA is the TdT-UTP DSB End Labeling (TUDEL) assay developed by Lutze et al. [41]. Like DI-PLA, the TUDEL labeling process begins via cell fixation and the application of T4 DNA polymerase blunting. However, instead of using BrdU as a nucleoside analogue and rolling circle amplification to produce a fluorescent signal, as per STRIDE, ethynyl-dUTP (EdU) is incorporated to blunted 3′-OH DNA ends and Huisgen cycloaddition (click chemistry) is used to produce a fluorescent signal. The incorporated EdU molecules contain reactive alkyne groups, which allow for the production of covalent bonds between EdU and a fluorescent dye (Figure 3C) [41]. 

While the TUDEL, STRIDE, and DI-PLA methodologies share some similarities, there are notable differences in their strengths, shortcomings, and validation. These assays all possess the ability for the direct in situ detection of DSBs in the nuclei of single cells, providing more accurate localization of breakage sites. Particularly, the DI-PLA and STRIDE techniques possess higher sensitivity compared to other DNA damage response markers due to incorporating rolling circle amplification reactions. This is best exemplified in the study that introduced STRIDE, where Kordon et al. were able to detect and precisely locate site-specific DSBs induced by CRISPR/Cas9 in the nucleus, demonstrating its ability to accurately assess even low levels of DNA damage [40]. Because of this, STRIDE is the most thoroughly validated of these assays, also supported by its comprehensive testing against various genotoxic agents, including doxorubicin, ultraviolet and laser micro-irradiation, hydrogen peroxide, and age-related and spontaneous DNA damage [40]. The establishment of DI-PLA and TUDEL was also compelling, both displaying the colocalization of γH2AX and 53BP1 with their DSB detectors post-irradiation [38,41].

Another notable difference is the use of T4 DNA polymerase in both DI-PLA and TUDEL, but not STRIDE (Table 2). As previously mentioned, T4 DNA polymerase is capable of filling in exposed 3′-OH ends of single-strand breaks and performing a 3′ overhang resection at sites of double-stranded breaks. In the case of DI-PLA, the purpose of end-blunting is to ligate biotinylated nucleotides at breakage sites via T4 ligase [38], and for TUDEL, to reduce non-specific background fluorescence by filling in single-strand breaks, thus reducing recognition by TdT [41]. TdT is capable of catalyzing oligonucleotides at the 3′-OH ends of either single- or double-strand breaks; however, it will preferentially bind to 3′-OH overhangs of the latter because of its catalytic characteristics [45,46,47,48,49]. According to Lutze et al., although SSBs are not an ideal substrate for TdT to conjugate oligonucleotides (DNA polymerase 1 is utilized in STRIDE when detecting SSBs for this reason [40]), substantial background fluorescence may be present, particularly at early timepoints after DNA damage induction [41]. This caveat would mainly affect STRIDE and TUDEL, both with a reliance upon TdT incorporation to detect DSBs, contributing to a lower signal-to-noise ratio when analyzing fluorescent foci. Moreover, the 3′-OH binding capacity of TdT means that there is also the ability for cytosolic DNA fragments and mitochondrial DNA breakages to be marked by STRIDE and TUDEL. Although DI-PLA utilizes proximal endogenous DDR markers that are associated with breakage sites to avoid these issues, the downregulation of repair machinery may lead to fewer detected breakage sites resulting in false negatives. Overall, the main limitations of these protocols are that although they can more accurately characterize DSBs, they are also prone to a degree of non-specificity and are susceptible to variation due to their complexity. Furthermore, the procedures to achieve such stains are elaborate (Figure 3D), making it difficult to troubleshoot and interpret results if issues arise.

As these DSB detection techniques are relatively cutting-edge, complex, and costly, there are not many published studies utilizing them for the quantification of DSBs. A 2021 thesis successfully reproduced the STRIDE technique to detect DSBs in both an adenocarcinoma line, MCF-7, and spermatozoa, after exposure to either ultraviolet light or hydrogen peroxide [50]. To date, no other publications cite the usage of DI-PLA or TUDEL experimentally. As it stands currently, the further use of these techniques is required to properly comment on their practicality and efficacy compared to other conventional and novel techniques alike. Regardless, the development of DI-PLA, STRIDE, and TUDEL comprises steps in the right direction for vastly improving the targeting of fluorescent foci to DSBs, in a bid to eliminate the ambiguity conferred by labeling endogenous factors, and to accurately study the mechanisms of DNA damage.

## 3. First Responders

First responder proteins are classified as proteins that arrive at the very first stages of DSB detection. These include proteins that are in the well-established conventional DDR, such as the MRN complex (composed of MRE11, RAD50, and NBS1) and Ku70/80, which promote HR and NHEJ repair, respectively, and members of the Sirtuin and poly (ADP-ribose) polymerase (PARP) families, which lie upstream of these pathways and act in promoting DNA repair. After the binding of this class of proteins to a DSB site, a cascade of DDR activation occurs (detailed in the “DDR Activators” subsection). Although they act via binding to the ends of DSBs akin to the exogenous DSB detectors, first responders are endogenous molecules and typically initiate or assist in the repair of DSBs, whereas DSB detectors do not. First responder proteins are ideal targets for visualization in instances where the initiation of DDR repair is concerned, as they are essential in both identifying DSB sites and amplifying downstream DDR factors. The rapid recruitment to sites of damage within seconds of an insult establishes them as DNA damage first responders, meaning early DDR kinetics can be observed by targeting these proteins.

### 3.1. PARP1

One of the first responders is PARP1, a member of the ADP-ribosyl transferase family responsible for initiating DNA damage repair. After a genotoxic insult, PARP1 rapidly localizes to DSBs within 0.5 s [22] and aids in the initiation of the DDR, capable of binding to DSBs via its zinc finger and tryptophan-glycine-arginine rich (WGR) domains [51]. Following this, it utilizes ADP-ribosyl enzymatic activity to remodel chromatin and recruit early DDR signaling proteins such as MRE11 and ATM to the breakage site, as well as PARP1-dependent transcription factors [52]. PARP1 binding motifs are also present within key HR genes, regulating proteins including breast cancer 1 (BRCA1) and 2 (BRCA2) [53,54], TOPBP1, which is involved in the recovery of stalled replication forks and ATR signaling [55], and OBFC2B and SSBIP1, which are components of the SOSS complex that participates in ATM signaling [56]. As such, PARP1 is a key promoter of the HR pathway in the S/G2 phase where it has been shown to remove Ku70/80 from DSBs [23]. 

**Figure 4:**
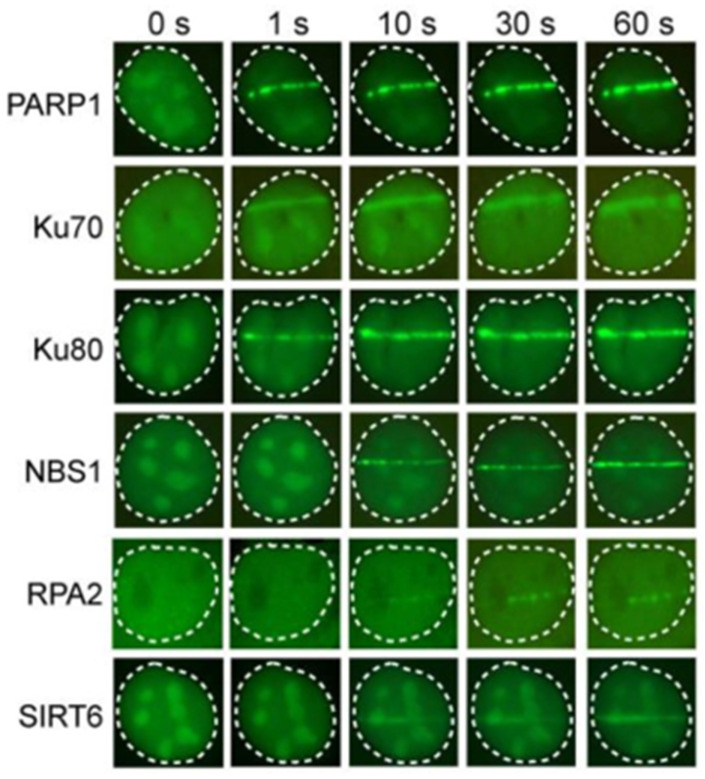
U2OS cells exposed to microirradiation, and kinetics monitored via live cell imaging. DDR proteins were tracked via transfecting GFP fusions of target proteins (i.e., PARP1-GFP, Ku70-GFP etc.) prior to the insult [23]. Reprinted with permission from Ref. [23]. 2018, Oxford University Press on behalf of the International Journal of Molecular Sciences.

Given its crucial role in DNA maintenance, there are several ways PARP1 can be detected. The first paper to document the association of PARP1 with MRE11, and by extension the MRN complex, was Haince et al. in 2008. Akin to the aforementioned Gam-GFP [14], they produced a PARP1-GFP vector to analyze the spatiotemporal accumulation of PARP1 in response to laser micro-irradiation in live cells [22]. Interestingly, they showed that PARP1 was necessary for the rapid recruitment of MRE11 and NBS1, where the recruitment of these factors was inhibited in PARP1-deficient cells. Additionally, transfection of the PARP1-GFP plasmid in PARP1-deficient cells increased the number of detectable 53BP1 foci post-insult, showing that cells expressing functional PARP1-GFP can recover DDR recruitment and downstream signaling [22]. More recently, Yang et al. established GFP vectors of PARP1 and various DDR factors, including the previously mentioned NBS1 within the MRN complex, Ku70/80, and SIRT6. From this, they could also determine the spatiotemporal recruitment kinetics of PARP1, where foci appeared 1 s after microirradiation (Figure 4) [23]. 

Although possessing multifaceted roles in the regulation of DSB repair, the sensitivity of PARP1 is not limited to DSBs. There is evidence that PARP1 can modulate the repair of SSBs including being implicated in the base excision repair pathway [57,58]. This includes the regulation of gene transcription [59], cell death [60,61,62], pro-inflammatory cytokines [63,64], and viral immunity [65]. It is a therapeutic target for cancer treatment due to this, with multiple PARP inhibitors currently approved for treatment of BRCA1/2 mutated breast, ovarian, prostate, and pancreatic cancers because of their tumoricidal properties [66,67]. There is also evidence that PARP1 is capable of activation via SIRT6 during oxidative stress [68], suggesting that its accumulation at damage sites is regulated depending upon specific cellular stresses. In a study conducted by Wang et al. utilizing immunofluorescence staining against PARP1, they demonstrated that PARP1 was capable of translocation into the cytoplasm following infection with herpes simplex virus type 1 (HSV-1) in HeLa cells [61]. When in some cases researchers might utilize this protein as an indication of DSB presence or repair initiation i.e., in response to chemical [69] or ionizing-radiation [70] induced DSBs, this is evidence of PARP1’s capacity to reside at intracellular locations distinct from DSBs. These factors may have unpredictable effects upon the localization of PARP1 foci, highlighting the need for careful interpretation of its presence at DNA damage sites or association with targets outside of the DDR. 

### 3.2. Ku70/80 and MRN

The MRN complex and Ku70/80 play critical roles in the repair of DSBs. That is, they are amongst the first proteins recruited to the site of a break, with there being evidence for them arriving at the site of damage within 10 s in the case of MRN [22,71], and 1 s for Ku70/80 [23]. The Ku70/80 complex is a heterodimeric protein that also binds to the DNA ends of DSBs but is predominantly involved in c-NHEJ. Binding the DSB in a ring-like structure that encapsulates duplex DNA [72], Ku mediates recruitment of the DNA-dependent protein kinase catalytic subunit (DNA-PKcs), which enables the synapsis of DSB ends and further recruitment of downstream c-NHEJ effector proteins [73]. The Ku complex also plays a role in telomere maintenance by protecting chromosome ends from degradation and recombination, localizing to telomeres through its affinity for the TRF1 shelterin protein, and ensuring genomic stability [74,75]. 

In contrast, the MRN complex consists of three subunits: MRE11, RAD50, and NBS1. MRE11 possesses endonuclease activity and processes DSBs by resecting the 5′ ends of the DSB, forming 3′ ssDNA overhangs on either side of the break to facilitate HR [76]. It is important to note that end resection is suppressed in the G1 phase of the cell cycle to promote NHEJ, while the converse is true within the S/G2 phases [77]. Prior to DNA replication in the early S phase, HR cannot engage as there is no sister chromatid from which complementary strands can be synthesized. Under these conditions, MRN also plays a role in canonical-NHEJ (c-NHEJ) and alternative-NHEJ (a-NHEJ) pathways [78], affecting mechanisms in lymphocytes including V(D)J [79] and class switch recombination [80]. Although its role is not yet clearly defined, producing mutations in the dimerization region of MRN has been shown to hinder NHEJ repair [81], where under normal conditions it is predicted to aid in the tethering of free DSB ends to facilitate c-NHEJ protein accumulation [82]. The RAD50 component facilitates the tethering of DNA ends through a zinc hook domain that stabilizes sister chromatids during HR and aligns broken ends during NHEJ [83], whilst NBS1 serves as a scaffold protein that recruits other repair factors to the DSB [84]. MRN also regulates the activity of telomerase [85,86], and activates ATM kinase [87], a key regulator of the DDR. 

Immunofluorescence assays are a common method for the detection of the MRN and Ku70/80 complexes. For MRN complex detection, antibodies against MRE11, RAD50, and NBS1 can be used to label DSB ends, and co-localization with downstream repair signals, such as γH2AX, a histone modification that is activated in response to DNA damage, can be used to further validate the recruitment of MRN to the breakage site. Similarly, antibodies against Ku70/80 can be used in conjunction with NHEJ repair factors, such as 53BP1, to validate its recruitment. Examples from previous studies include Mirzoeva et al., who applied the indirect immunofluorescent marking of MRE11 after cells were exposed to gamma irradiation [88], and Britton et al., who applied the same technique for Ku70 in response to laser micro-irradiation [89]. An alternative fluorescence detection method is by producing vectors that encode the MRN or Ku fused with fluorescent proteins for transfection, Yang et al. using this technique to analyze their repair kinetics alongside PARP1 [23]. It should be noted that even though both MRN and Ku70/80 are regarded as sensors of DSBs and are capable of binding to DSB ends, there are instances where they may not bind to all breaks present within a cell. For example, in situations where Ku70/80 is displaced during HR, particularly in the S/G2 phases of the cell cycle, foci will not be resolved at each DSB end if only Ku is utilized as a quantifier of DSB ends. Therefore, although MRN and Ku70/80 predominantly play important roles in DSB recognition and repair, their functions differ in terms of the specific repair pathways they promote and in which they are involved. For instance, there is evidence of both MRE11 [90] and Ku80 [91] present within mitochondria. As mitochondrial DNA undergoes distinct DSB repair processes when compared to nuclear DNA, in addition to HR and c-NHEJ [92], this can ultimately complicate the interpretation of immunofluorescence data. Nuclear foci may thus provide more meaningful interpretations when trying to interpret cellular responses to genotoxicity. Factors that promote the accumulation of MRN and Ku70/80 to DSBs (e.g., PARP1) also have the potential to influence their kinetics. This limits their usage as a one-to-one marker of DSBs due to this degree of endogenous variability, but they are extremely useful in understanding which proteins associate with or regulate their accumulation at breakage sites. Regardless, immunofluorescence or GFP-fusion proteins can be used to detect and analyze the recruitment and dynamics of both complexes in response to damage, which can provide valuable insights into their functions in DSB detection and repair.

### 3.3. SIRT6

**Figure 5:**
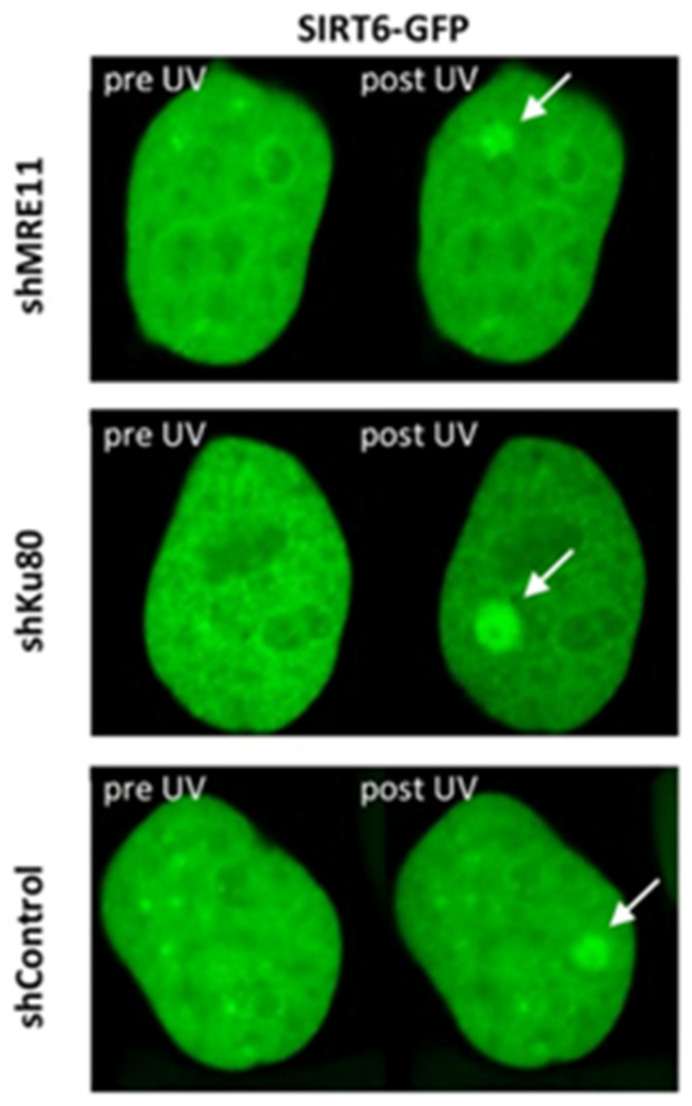
HeLa cells exposed to UV laser irradiation and recruitment kinetics of SIRT6-GFP monitored via live cell imaging. Prior to irradiation, key DDR proteins were silenced, including MRE11 (shMRE11) and Ku80 (shKu80), and compared to HeLa cells without silencing (shControl). This evidence supports that SIRT6 activation is not dependent on DSB detectors within the DDR [97]. Reprinted with permission from Ref. [97]. 2020, eLife under the CC BY 4.0 Deed|Attribution 4.0 International | Creative Commons.

Sirtuins are a family of regulatory proteins responsible for maintaining genomic stability. They possess a variety of signaling capabilities, including cell cycle control, epigenetic chromatin modification, mitochondrial metabolism, and the DDR. As such, dysfunctions in Sirtuin class proteins are responsible for a variety of diseases related to aging, obesity, and cancer progression [93]. The downregulation of SIRT6 has been implicated in increased rates of neurodegeneration, aging, cancers, and tissue degradation due to its importance in recognizing DNA damage [94,95,96]. Onn et al. were the first to report SIRT6 as a DSB sensor in 2020, establishing its role in the initiation of the DDR. To explore this, they produced multiple SIRT6 plasmids, including SIRT6-GFP and -RFP, to validate its involvement in the DDR [97]. One of the unique attributes that they discovered is its ability to rapidly localize to DSB sites independently from DNA damage sensors within the DDR pathway (Figure 5). SIRT6 was previously reported, by Toiber et al., to accumulate at DSBs within 5 s of a DSB, with its accumulation plateauing at 30 s post-insult [96]. Inhibiting key factors of the DDR pathway, including γH2AX or 53BP1, had no impact on SIRT6 initiating the repair response [97]. 

SIRT6 contains a core DNA-binding domain that binds to SSBs via a tunnel-like tertiary structure, surrounding broken DNA strands to prevent further DNA damage, and facilitate the recruitment of DDR factors. This core DNA-binding domain is conserved amongst Sirtuin family proteins, sharing the DNA-binding capability of SIRT6, albeit with decreasing affinity. In addition to binding to SSBs, electrophoretic mobility shift assay results suggest that cooperative binding allows SIRT6 to bind to open-ended DSBs in a dimer conformation. Silencing SIRT6 appears to affect the expression of downstream signaling factors within the DDR pathway, including the phosphorylation of H2AX and recruitment of ATM, Ku70/80, BRCA1, and 53BP1, thus suppressing the initiation of both HR and NHEJ pathways [97]. In using SIRT6 as a DSB detector, the development of vectors expressing fluorescent SIRT6 that emit distinct signals upon dimerization, including Förster resonance energy transfer (FRET) fluorophores [98], may be a viable solution to increase foci specificity. This would effectively eliminate the off-target binding of SIRT6 to SSBs and produce foci specific to DSBs, given the assumption that SIRT6 exclusively binds to DSBs in a dimer configuration.

## 4. DDR Activators

**Figure 6:**
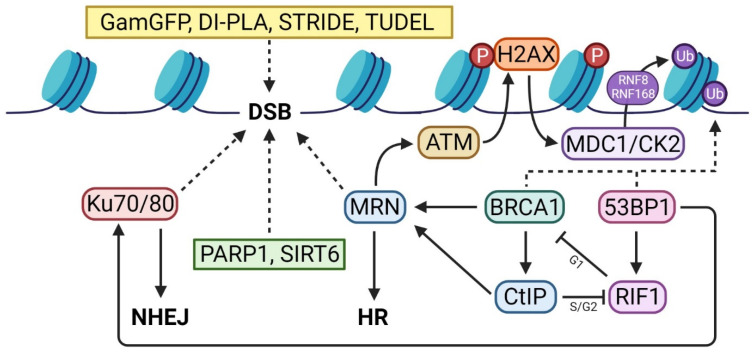
Protein interaction chart of DSB detectors and repair pathway decision process. Immunofluorescent markers of DSBs, including DI-PLA, STRIDE, and TUDEL, act independently of the DSB repair process when binding to breakage sites. SIRT6 and PARP1 are also capable of arriving at breaks independently of DDR activators. Decision-making between HR and NHEJ is driven by antagonism between BRCA1 and 53BP1 depending on cell cycle phase.

DDR activators are proteins that propagate the cellular response to DNA damage, downstream of the first responder molecules that accumulate at DSB sites. After the MRN complex detects a DNA lesion, autophosphorylated ATM monomers are recruited to the breakage site [76]. Chromatin-based DSB signaling is then initiated, with ATM rapidly phosphorylating Ser-139 on histone variant H2AX, resulting in the formation of γH2AX. The phosphorylation of this subunit acts as an epigenetic signal indicative of the presence of DSBs, which is recognized by mediator of DNA damage checkpoint protein 1 (MDC1). The constitutive phosphorylation of MDC1 by casein kinase 2 (CK2) and chromatin-bound ATM initiates a positive feedback loop, whereby additional MRN and ATM are recruited to damage sites [99]. Phosphorylated MDC1 then recruits the E3 ubiquitin ligases RING finger 8 (RNF8) and RNF168, which ubiquitylate chromatin surrounding the break [100]. This promotes remodeling that facilitates access of scaffolding and repair proteins to the DSB site, in addition to providing binding sites for downstream repair machinery [101,102]. After this point, the pathway diverges into the two predominant DSB repair pathways: HR and NHEJ (Figure 6) [11,103,104].

Currently, the most widely adopted markers used to quantify DSB insults are the aforementioned DSB activators, the most popular of these being γH2AX. With the phosphorylation of histone variant H2AX being one of the cells earliest steps in initiating the DDR after the induction of DSBs, it has been widely utilized as a cellular marker of the presence of these lesions via immunofluorescent labeling, with discrete foci at or in proximity to DSBs. This allows for the visualization and quantification of DDR activation, caused by ionizing radiation or chemical agents, by using fluorescence microscopy. As ATM and MDC1 function in the initiation of the DDR, they may also be used as effective stains alongside the well-established γH2AX as markers for the activation of DSB repair. However, out of all DDR activators, γH2AX has been favored due to its convenience, efficacy, and sensitivity [105].

### 4.1. ATM

ATM, a member of the PI3K-like protein kinase family, plays a vital role in various cellular functions, such as cell-cycle regulation, chromosome condensation, and DNA maintenance. Its key function involves transmitting signals after the recognition of DSBs, thus propagating the cascade of damage recognition and facilitating the recruitment of DDR proteins to the DSB site. Under conditions where the cell is not under stress, ATM exists in an inactive homodimeric conformation [106]. However, upon exposure to genotoxic stress, ATM dissociates from its dimeric structure through autophosphorylation at several sites, including Ser367, Ser1893, Ser1981, and Ser2996, in addition to acetylation on Lys3016. From here, it regulates multiple canonical (HR/NHEJ) and non-canonical repair pathways, which encompasses hundreds of downstream effector molecules within the DDR [107]. 

Fluorescence assays utilized in the detection of ATM employ similar techniques to the marking of upstream DDR factors mentioned previously. Davis et al. used ATM tagged with yellow fluorescent protein (YFP-ATM) to determine the real-time recruitment and kinetics of ATM in response to laser micro-irradiation in live cells [26]. A vector containing the YFP-ATM fusion protein was transiently expressed in AT5 cells, which were then exposed to laser micro-irradiation. The accumulation of ATM at damage sites was recorded at multiple time points over 2 h, with a peak relative fluorescence intensity at 20 min post-insult [26]. Ray et al. sought to discover additional proteins that promote ATM recruitment in response to UV-irradiation-induced damage. Using immunofluorescence, they found that ATM is regulated by nucleotide excision repair proteins during G1, but not during the S phase [108], emphasizing the importance of the cell cycle for DDR factor activation.

**Figure 7:**
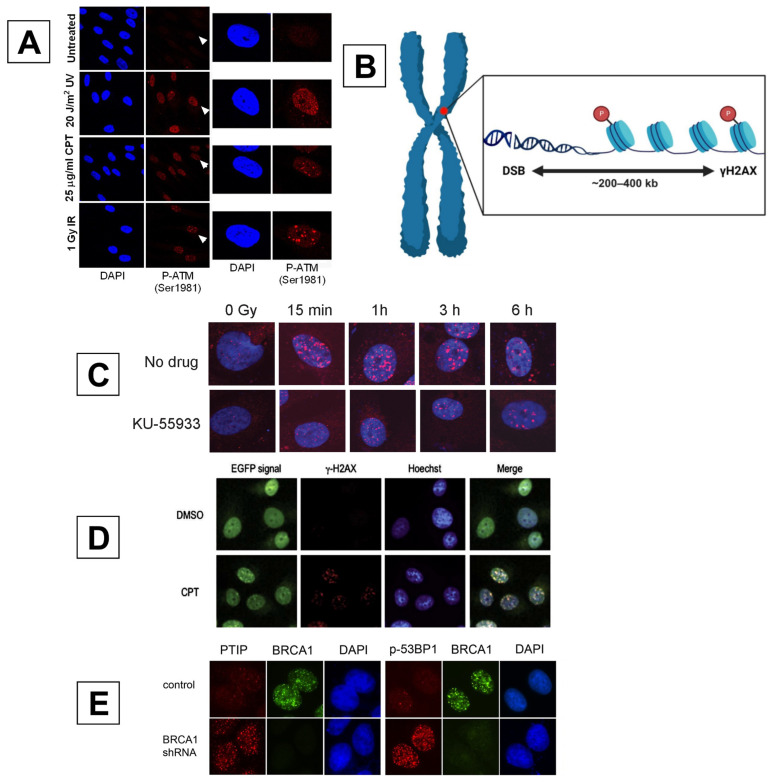
(**A**) Immunofluorescence images of quiescent human dermal fibroblasts 1 h after exposure to UVC, camptothecin, or 1 Gy ionizing radiation (source not mentioned). Cells were stained with an anti-phospho-ATM (1981) antibody (red) and DAPI (blue) [111]. Reprinted with permission from Ref. [111]. 2015, Springer Nature. (**B**) Visual representation of the spatial arrangement of γH2AX foci relative to DSB sites (not to scale). Occurring proximally to beaks within the initial stages of detection, thousands of H2AX phosphorylation sites can rapidly spread to up to 200–400 kb from the site of a DSB [27,113,114]. Because of this, foci are capable of being resolved at nuclear locations distinct from DSBs during immunofluorescent visualization. (**C**) Adams et al. highlight the dependence of H2AX phosphorylation on ATM through the use of the ATM inhibitor KU-55933. Immunofluorescence images of astrocytes pre-treated with 10 µM KU-55933 for 1 h, followed by 2 Gy radiation. The reduced foci count indicates reduced DSB repair and not a reduction in the formation of DSBs. Cells were fixed after the radiation insult and stained with an anti-γH2AX antibody (red) and DAPI (blue) [115]. Reprinted with permission from Ref. [115]. 2010, PLoS One under the CC BY 4.0 Deed | Attribution 4.0 International | Creative Commons. (**D**) EGFP-MDC1-expressing MCF7 cells exposed to either 0.1% (*v*/*v*) DMSO or 10 µM calprotectin (CPT) for 1 h. After treatment, cells were fixed and stained with an anti-γH2AX antibody (red) and Hoechst (blue), alongside the expression of EGFP-MDC1 (green). Co-localization between EGFP-MDC1 and γH2AX is depicted in yellow (Merge) [116]. Reprinted with permission from Ref. [116]. 2014, Genes and Environment under the CC BY 4.0 Deed | Attribution 4.0 International | Creative Commons. (**E**) HeLa cells synchronized in the S/G2 phase via double-thymidine block irradiated with 10 Gy γ-radiation and infected with control or BRCA1 short hairpin RNA (shRNA). Cells were fixed 2 h post-irradiation. Left: stained with anti-PTIP (red) and anti-BRCA1 antibodies (green) and counterstained with DAPI. Suppression of BRCA1 in the S/G2 phase restored the expression of PTIP. Right: stained with anti-p53BP1 (red) and anti-BRCA1 (green) antibodies, counterstained with DAPI. The presence of BRCA1 inhibits the formation of 53BP1 foci, and the suppression of BRCA1 through shRNA restores 53BP1 foci formation [117]. Reproduced with permission from Ref. [117]. 2015, Springer Nature under the CC BY 4.0 Deed|Attribution 4.0 International|Creative Commons.

There are situations where ATM can localize differently in response to various cellular stressors. When ATM is activated by DSB formation, it results in the localization of activated ATM, which appears as individual fluorescence foci within the nucleus and is associated with DNA damage repair machinery [109]. However, in a study conducted by Bakkenist et al. in 2003, it was found that ATM was still capable of phosphorylation even after cells were exposed to stressors that did not cause DSBs [106]. In the case of non-DSB-inducing stressors, including topoisomerase inhibitor and hypotonic buffers, the activated ATM appears as phosphorylated ATM-Ser1981 scattered throughout the nucleus in a diffuse pattern without forming discrete foci (Figure 7A) [109,110,111]. It has been proposed that this is because ATM activation is not strictly dependent upon the direct binding of ATM to DSBs or the MRN complex [112]. This should be considered prior to the analysis of ATM via fluorescence-based techniques, as it will ultimately influence its intracellular distribution when observed via microscopy.

### 4.2. γH2AX

In response to a DSB, numerous chromatin modifications occur to initiate repair, rapidly and meticulously. One of the most important of these is the phosphorylation of histone variant H2AX at Ser-139 by ATM (as well as DNA-PKcs and ATR) to form γH2AX [104]. This promotes the recruitment of downstream DSB repair molecules, in addition to ensuring genomic stability [118]. However, there are common misconceptions when utilizing proteins within the DDR as markers for DSBs, as they are only an indication of whether DNA repair has been initiated, and not markers of where DSBs are located specifically [12]. Regarding γH2AX, it is generally true that the presence of DSBs induces the phosphorylation of H2AX. Yet this is often misinterpreted in the reverse case, with the assumption that all γH2AX foci represent DSBs. Although γH2AX has been cited for its reliability, many inconsistencies have been reported regarding the quantitative relationship between γH2AX foci production and DSBs [13]. These include the time taken to detect the maximum number of γH2AX foci via microscopy being 15–30 min post-irradiation [103], the phosphorylation of H2AX at sites other than where DSB repair is occurring [119] (Figure 7B), and the clustering of repair foci [120]. 

In mitotic cells, there also appears to be the damage-independent phosphorylation of H2AX, suggesting that γH2AX performs functions outside of signaling DSB repair [121]. Finally, the loss of γH2AX foci is assumed to be the result of complete DSB repair. Whether this is triggered by ligation of the sugar-phosphate backbone and the re-annealing of DNA strands or by chromatin reverting to its normal conformation is unclear. However, Bouquet et al. highlight that there are still questions surrounding the disappearance of γH2AX foci and its linearity to the degree of the genotoxic insult [122,123]. Exposure to severe insults may result in a persistent, saturated γH2AX signal, allowing for the continued detection of γH2AX foci after DSBs have been fully repaired [122]. They also show that TGFβ inhibition prior to radiation treatment reduces γH2AX foci formation [123], highlighting that impeding the pathways that regulate DDR activation has a substantial impact on foci formation. All of these factors may alter the total counts of γH2AX foci and therefore misrepresent the true number of DSBs present. For example, if DSB sensors or upstream repair activators, such as ATM are inhibited (Figure 7C) or downregulated, γH2AX will not form to its fullest extent despite the existence of DSBs. However, this does not discredit the value in conducting these types of assays in other contexts. For instance, γH2AX is extremely valuable in assessing the extent of DDR activation (or inhibition) in a pathway-independent fashion, which is essential for understanding how regulation of the DDR may be affected in response to a genotoxic insult. This also allows for comparisons to be drawn between the number of DSBs produced by a genotoxic insult and the extent that the DDR is activated in response to these breakages. Making this distinction is pivotal to prevent misinterpretation of radiobiological data: that these DDR activators should be utilized as markers in the analysis of DDR activation kinetics, and not in the quantification of either DNA breakage formation at the DNA level or complete repair.

### 4.3. MDC1

Another key regulator within the DDR, MDC1, is activated within 20–30 s of DNA damage [24]. It achieves this through ATM-dependent-phosphorylation, as well as specific recognition of γH2AX’s C-terminus via its BRCA1 carboxy-terminal (BRCT) tandem domains [124,125]. One of the main roles of MDC1 within the cellular response to DSBs is to amplify the phosphorylation of γH2AX proximal to breakage sites [113]. MDC1 also facilitates the binding of the ubiquitin ligase RNF8 to chromatin, allowing for ubiquitylation and the recruitment and turnover of downstream effectors that regulate DSB repair and pathway choice [126]. These include associations with MRE11 and NBS1 within the MRN complex [24], RAD51 [127], PALB2 [126], and the repair choice mediators BRCA1 and 53BP1 [124]. Described as a “master regulator” of the DDR, MDC1 promptly recruits a variety of factors to DBSs. This facilitates efficient HR and NHEJ repair and enhances the signaling of damage to neighboring chromatin, thereby ensuring genomic stability [128]. Lee et al. established a link between inhibitor of DNA-binding 3 (ID3) and MDC1, finding that there is an ID3–MDC1-dependent interaction required for MDC1 accumulation at sites of DSB repair. The depletion of ID3 resulted in impaired MDC1 recruitment at DSB sites, also resulting in a reduction in γH2AX-bound MDC1 foci and cellular sensitization to radiation [128]. This has broad implications when choosing MDC1 as a candidate marker, as its expression modulates the accumulation of DDR proteins at DSB sites.

Plasmids encoding fluorescent MDC1 have been produced to analyze its recruitment dynamics and association with endogenous DDR factors. Matsuda et al. utilized a vector containing enhanced green fluorescent protein (EGFP)-fused to MDC1 to visualized its accumulation within MCF7 cells exposed to multiple mutagens, including camptothecin, cisplatin, and DNA-adduct-forming compounds. To validate whether the normal function of MDC1 was compromised when fused to EGFP, the nuclear location of EGFP-MDC1 foci was compared to the staining of MDC1 with anti-MDC1 and anti-γH2AX antibodies. After EGFP-MDC1/MCF7 cells were exposed to 10µM camptothecin treatment for 1 h, fluorescence imaging revealed that the localization of EGFP foci overlapped with both anti-MDC1 and anti-γH2AX signals, suggesting that EGFP-MDC1 maintained its functional capability to associate with DSB sites after exposure to DNA damage (Figure 7D) [116]. 

One notable limitation in this experimental protocol was that the proportion of EGFP-MDC1-expressing cells was 1%, meaning that, in this instance, high-throughput analysis would present difficulties [116] Moreover, there appears to be a high background level of EGFP, resulting in poor sensitivity and preventing the detection of low levels of MDC1. Similarly, Lukas et al. produced GFP and YFP fusion proteins of MDC1. They discovered that NBS1 recruitment at DSB sites was heavily reliant upon the presence of MDC1. In cells exposed to MDC1-targeting siRNAs, NBS1 exhibited a reduced affinity to bind and remain at damaged chromatin [24]. Although acting chronologically after the binding of MRN to DSBs or is considered “downstream” of DSB sensing, MDC1 acts in assisting nearly every stage of the response to DNA damage. Because MDC1 regulates a variety of DDR proteins, it is an ideal marker for a wholistic overview of repair recruitment. However, contrasting γH2AX, its damage kinetics do not possess a defined relationship amongst all cell types and all pathways of DSB repair [129], limiting its utility in the quantification of genotoxicity. 

## 5. Repair Pathway Specific Assays

The progression towards either HR or NHEJ is controlled by several factors. The error-free DNA repair process HR is the predominant mechanism of repairing DSBs, but only in the S and G2 phases of the cell cycle. It is capable of fully restoring damaged genomic sequences; however, it requires sister chromatids to utilize as a template for replication. In contrast, NHEJ repair is error-prone as it is performed with no regard to homology, yet it is the most frequently utilized DSB repair mechanism in eukaryotes. While predominantly employed in the G0, G1, and early S phase, NHEJ can occur during any time in the cell cycle. HR is characterized by extensive end resection at DSB sites; in contrast, end protection typifies the selection of NHEJ [130], ultimately influencing the set of proteins present at each DSB site depending on which pathway will proceed. Changes in cell cycle distribution consequently change the portion of cells undergoing either HR or NHEJ repair and thus affect the proportion of proteins expressed in either of these pathways.

### BRCA1 and 53BP1

In conjunction with cell cycle dependence and end resection, one model posits that the choice between HR and NHEJ pathways is determined by the action of two key proteins after chromatin ubiquitylation [131], these being the recruitment of either BRCA1 to promote HR [132,133] or 53BP1 for NHEJ [134,135]. BRCA1 and 53BP1 interact antagonistically, with the activation of one inhibiting the other’s ability to associate with damaged chromatin, making them important for determining which DSB repair pathway will proceed [131,136].

After RNF8- and RNF168-induced chromatin ubiquitylation, the progression towards NHEJ is initiated through the subsequent ubiquitylation of H2AK15, which promotes the recruitment of 53BP1 and the exposure of H4K20me2 residues [102,135]. 53BP1 possesses various functions in the recruitment of additional DSB-repair proteins, checkpoint signaling, and NHEJ initiation [102,135,137]. Another potential substrate of RNF8 and RNF168 is the BRCA1–Abraxas–RAP80 complex, with RAP80-containing ubiquitin-interaction domains. This allows BRCA1 to form the RING heterodimer [138], and consequently associates with C-terminal binding protein-interacting protein (CtIP) and the MRN complex to promote 5′ resection at the DSB ends, forming 3′ ssDNA overhangs on either side of the break [139,140,141]. To prevent progression towards NHEJ during S and G2 phases, studies indicate that BRCA1 is capable of displacing 53BP1 and its associated effector protein, RAP1-interacting factor 1 (RIF1), from damaged chromatin [142,143,144]. The mechanism of action responsible for this is unclear; however, the result is extensive end-resection that drives the recruitment of downstream HR factors [145]. During G1, the opposite occurs, and the end-resection-promoting activity of BRCA1 is inhibited by the 53BP1–RIF1 complex, limiting the accumulation of BRCA1 at DSB sites, as well as the association with the CtIP-MRN complex. 53BP1’s mechanism of action includes blocking end-resection via recruitment of the Shieldin (SHLD) complex [146], as well as localized fill-in DNA synthesis through CTC1–STN1–TEN1 (CST) and DNA polymerase α (Pol α) to reduce the extent of resected DSBs [147]. This allows for the predomination of NHEJ, as end resection is severely impeded. 

Feng et al. used antibodies against 53BP1 to analyze its association with the downstream effectors RIF1 and Pax transactivation domain-interacting protein (PTIP) [117]. They produced a highly specific PTIP antibody that recognized it as a pan-nuclear protein in undamaged HeLa cells. However, after exposure to γ-irradiation, PTIP could form discrete foci. Interestingly, an analysis of cells irradiated at different stages of the cell cycle revealed that PTIP and BRCA1 exhibited a mutually exclusive relationship when forming foci and that both ATM-dependent 53BP1 phosphorylation and PTIP and RIF1 accumulation at DSB sites is limited to the G1 phase [117], suggesting that PTIP contributes to the inhibition of HR (Figure 7E).

Another study by Wei et al. utilized GFP-fused BRCA1 to analyze its in situ response to microirradiation, revealing that the N-terminal domain of BRCA1 is associated with Ku80, in addition to showing that missense mutations within cancer cell lines substantially altered the repair kinetics of BRCA1 [28]. This highlights that the recruitment of pathway-specific factors is not only limited to the cell phase, but also cell type, which is pertinent to consider when making conclusions about their interactions with other key DDR components.

As the interactions by which BRCA1/53BP1 antagonism occurs have not yet been thoroughly validated, leaving some ambiguity in the steps that precede the DSB repair pathway choice, an analysis of proteins at this stage can provide information into the time and duration that these proteins associate with breakage sites and the extent of their activation, in addition to identifying proteins of interest that they may co-localize with. Therefore, they are prime candidates for immunofluorescence imaging to determine cellular progression towards resolving DSBs through HR or NHEJ, enabling a quantitative foci comparison to upstream DSB detectors, DDR activators, and downstream HR and NHEJ factors. Moreover, they can be utilized alongside other techniques, such as the Single Molecule Analysis of Resection Tracks (SMART) assay, to determine the extent of end-resection during the repair of DSBs through HR works [148]. SMART functions by incorporating nucleotide analogues (such as 5-Ido-2′-deoxyuridine, IdU) to cells during the S-phase, followed by cell lysis and the stretching of DNA fibers on a microscope slide. Immunofluorescence staining is then used to stain the nucleotide analogues incorporated into the stretched 3′ single-stranded DNA strands. Fluorescence microscopy and image analysis packages can then be used to determine the lengths of these DNA strands. This is useful in measuring the extent of DNA end-resection in response to a genotoxic insult and, by extension, the efficacy of end-resection machinery [148]. This can allow for clearer relationships to be identified between the activation/inhibition of the DDR (e.g., PARP1 inhibitors) [139], how BRCA1 and 53BP1 expression is affected by changes in repair regulation, and if this results in cells preferring one DSB repair mechanism over another.

## 6. Conclusions

The appearance of a wide variety of new and innovative fluorescence-based DSB detection techniques is promising, continually pushing the envelope in improving characterization of DNA damage and responses. Moreover, there are a variety of targets to selectively analyze the regulation of specific stages of the DSB-induced DDR. Fluorescently conjugated proteins, such as Gam-GFP and SIRT6-GFP, are prime examples of simple, single-step biomolecular markers for DSBs. The interference between Gam-GFP and endogenous Ku proteins and the ability of PARP1 and SIRT6 to also bind to SSBs are issues that must be overcome for these proteins to be established as robust DSB markers. STRIDE, TUDEL, and DI-PLA also appear to present these shortcomings, yet due to the relative novelty of these methodologies, information on their reliability is sparse. More widespread utilization and optimization of these new methodologies is required before establishing them as “best-in-class: DSB detectors. Endogenous factors responsible for signaling the accumulation of DSB repair machinery, including PARP1, MRN, ATM, and the commonly utilized γH2AX, are invaluable markers in providing an overview of widespread DSB repair activation. This can be applied when comparing DSB and DDR markers, particularly in studies contrasting the kinetics of DSB formation versus repair activation. Although the mechanism of action by which HR/NHEJ promotion has not yet been fully established, BRCA1 and 53BP1 stand out as proteins that bias each pathway, respectively, and indicate the DSB repair pathway choice. Overall, due to the complexity of the DDR, careful consideration is required to select the correct target for the stage of DSB repair being studied, also accounting for multifunctional proteins that are not confined to operating within DSB detection and repair alone. Utilizing each of these DSB-DDR targets in a wide variety of cell lines and insults, whilst drawing comparisons between them, is the only way to ascertain which are most valuable in characterizing each stage of DSB repair. The continual development of accurate markers for each repair stage is essential not only in providing more accurate data in response to cytotoxic damage, but also in gaining a greater understanding of the mechanisms that define the DNA damage response overall.

## Figures and Tables

**Table 1:** Summary of the DNA damage and repair assays reviewed. Stars ★ indicate the relative level of complexity of each assay, with five stars being the most complex.

DDR Stage	Marker	Complexity	Uses	Challenges
DSB Detector Assays	GamGFP	★★★☆☆	Binds specifically to DSBs. Simple protocol compared to other DSB assays.	Competition with endogenous Ku in mammalian cells. Optimizing transfection efficiency.
	DNA damage In situ ligation followed by Proximity Ligation Assay (DI-PLA)	★★★★★	Identifies DDR proteins proximal to DSB sites.	May not target all DSBs due to changes in DDR activity.
	SensiTive Recognition of Individual DNA Ends (STRIDE)	★★★★★	Highly sensitive. Detects single CRISPR/Cas9 nicks.	TdT enzyme may result in non-specificity (SSBs, cytosolic DNA, mitochondrial DNA)
	TdT-UTP DSB End Labeling (TUDEL)	★★★★☆	High sensitivity.	
First Responders	Poly (ADP-ribose) Polymerase-1 (PARP1)	★★★☆☆	Active 0.5 s post-insult. Recruits multiple DDR factors.	Can reside at intracellular locations distinct from DSBs.
	Ku70/80	★★☆☆☆	Active 1 s post-insult. Involved in canonical-NHEJ (c-NHEJ).	Displaced from DSBs during S/G2 phases and HR.
	Sirtun 6 (SIRT6)	★★★☆☆	Active 5 s post-insult. Binds to DSBs independently of other DDR factors.	Binds to SSBs. FRET to identify dimer pairs at DSB sites.
	MRN	★★☆☆☆	Active 10 s post-insult. Involved in HR.	Involved in c-NHEJ and alternative-NHEJ (a-NHEJ), role not yet established.
DDR Activators	Ataxia-Telangiectasia Mutated (ATM)	★★☆☆☆	Key DDR regulator. Phosphorylates H2AX.	Activity is cell-cycle-dependent. Non-DSB damage can activate ATM, do not form discrete foci.
	γH2AX	★★☆☆☆	Well-established DDR marker. Activated to promote DSB repair molecules.	Foci do not always indicate the presence or proximity of DSBs. Activation kinetics vary.
	Mediator of DNA Damage Checkpoint protein 1 (MDC1)	★★☆☆☆	Enhances signaling of damage. Regulates multiple molecules across most stages of repair.	Damage kinetics can be inconsistent across cell types due to regulating multiple proteins.
Repair Pathway Specific Assays	Breast Cancer 1 (BRCA1)	★★★☆☆	Regulates HR initiation.	BRCA1 and 53BP1 antagonism. Repair kinetics altered in different cell types (e.g., cancers).
	p53 Binding Protein 1 (53BP1)	★★★☆☆	Regulates NHEJ initiation.	

**Table 2:** Comparative table highlighting the key differences among the DI-PLA, STRIDE, and TUDEL DSB detection assays. It outlines the main steps of each protocol in chronological order, left to right, from whether cells are treated with T4 DNA polymerase through to fluorescence-based detection.

Technique	T4 Pol Blunting	Incorporation Reagent	DSB End Conjugate	Antibody Pair	Signal Amplification	Fluorescent Signal
DI-PLA	Yes	T4 Ligase	Biotin linker	Biotin + proximal DDR marker	Yes	Proximity Ligation Assay
STRIDE	No	TdT	BrdU	BrdU + BrdU	Yes	Proximity Ligation Assay
TUDEL	Yes	TdT	EdU	None	No	Azide-linked Fluorophore

## Data Availability

Data sharing not applicable. No new data were created or analyzed in this study. Data sharing is not applicable to this article.
